# A tertiary approach to improving equity in health: quantitative analysis of the Māori and Pacific Admission Scheme (MAPAS) process, 2008–2012

**DOI:** 10.1186/s12939-015-0133-7

**Published:** 2015-01-20

**Authors:** Elana Curtis, Erena Wikaire, Yannan Jiang, Louise McMillan, Rob Loto, Airini, Papaarangi Reid

**Affiliations:** Te Kupenga Hauora Māori, Faculty of Medical and Health Sciences, University of Auckland, Private Bag 92015, Auckland, New Zealand; Department of Statistics, Faculty of Science, University of Auckland, Private Bag 92015 Auckland, New Zealand; Faculty of Human, Social and Educational Development, Thompson Rivers University, Kamloops, British Columbia Canada

**Keywords:** Māori, Pacific, Indigenous, Ethnic minority, Health workforce development, Tertiary admission, Multiple mini interview, Widening participation, Secondary education

## Abstract

**Introduction:**

Achieving health equity for indigenous and ethnic minority populations requires the development of an ethnically diverse health workforce. This study explores a tertiary admission programme targeting Māori and Pacific applicants to nursing, pharmacy and health sciences (a precursor to medicine) at the University of Auckland (UoA), Aotearoa New Zealand (NZ). Application of cognitive and non-cognitive selection tools, including a Multiple Mini Interview (MMI), are examined.

**Methods:**

Indigenous *Kaupapa Māori* methodology guided analysis of the Māori and Pacific Admission Scheme (MAPAS) for the years 2008–2012. Multiple logistic regression models were used to identify the predicted effect of admission variables on the final MAPAS recommendation of *best starting point* for success in health professional study i.e. ‘CertHSc’ (Certificate in Health Sciences, bridging/foundation), ‘Bachelor’ (degree-level) or ‘Not FMHS’ (Faculty of Medical and Health Sciences). Regression analyses controlled for interview year, gender and ancestry.

**Results:**

Of the 918 MAPAS interviewees: 35% (319) were Māori, 58% (530) Pacific, 7% (68) Māori/Pacific; 71% (653) school leavers; 72% (662) females. The average rank score was 167/320, 40–80 credits below guaranteed FMHS degree offers. Just under half of all interviewees were recommended ‘CertHSc’ 47% (428), 13% (117) ‘Bachelor’ and 38% (332) ‘Not FMHS’ as the best starting point. Strong associations were identified between Bachelor recommendation and exposure to Any 2 Sciences (OR:7.897, CI:3.855-16.175; p < 0.0001), higher rank score (OR:1.043, CI:1.034-1.052; p < 0.0001) and higher scores on MAPAS mathematics test (OR:1.043, CI:1.028-1.059; p < 0.0001). MMI stations had mixed associations, with academic preparation and career aspirations more consistently associated with recommendations.

**Conclusions:**

Our findings raise concerns about the ability of the secondary education sector to prepare Māori and Pacific students adequately for health professional study. A comprehensive tertiary admissions process using multiple tools for selection (cognitive and non-cognitive) *and* the provision of alternative entry pathways are recommended for indigenous and ethnic minority health workforce development. The application of the MMI within an equity and indigenous cultural context can support a holistic assessment of an applicant’s potential to succeed within tertiary study. The new MAPAS admissions process may provide an exemplar for other tertiary institutions looking to widen participation via equity-targeted admission processes.

## Introduction

There are well-documented health inequities for Māori, the indigenous peoples of Aotearoa New Zealand (NZ) and Tagata Pasifika (‘Pacific’) a heterogeneous composite of peoples with Pacific nation ancestry born and living in NZ. Evidence identifies lower life expectancy, higher rates of disease and disability, and a reduced likelihood of receiving high quality hospital healthcare for Māori and Pacific compared to non-Māori non-Pacific peoples [1-5].

Achieving health equity for indigenous and ethnic minority populations requires multi-level structural change across broad health contexts and related sectors [6]. A key component in achieving health equity for Māori and Pacific peoples includes the development of a diverse health workforce that reflects the population and society it aims to serve [7]. Māori make up 14.9% of the NZ population but only 3.1% of doctors, less than 1.4% of pharmacists, 2.9% of dentists and 6% of nurses [8,9]. Pacific peoples make up 7.4% of the NZ population but only 1% of doctors, 0.2% of pharmacists, 0.6% of dentists and 2.2% of nurses [10-12]. Increasing the number of indigenous and ethnic minority health professionals has been hypothesised to: increase healthcare delivery to under-represented and low-income populations [7,13]; enhance patient satisfaction associated with patient preference for ethnically-concordant physician interactions [14,15]; and, potentially reduce physician bias that can contribute to ethnic inequities in access to high quality healthcare services [16].

A focus on Māori and Pacific health workforce development is also driven by general workforce demand pressures [7,17-20]. Because Māori and Pacific are young, high-growth, population groups there will be an increasing future reliance on the Māori and Pacific working population to support the economy and ageing of the non-Māori non-Pacific population [21,22]. This approach aligns with calls to reduce NZ’s recent reliance on overseas-trained health practitioners by growing a health workforce sourced from NZ communities [23,24].

Perhaps more importantly, increasing Māori and Pacific access to higher-level educational qualifications allows direct benefits from the higher incomes and opportunities associated with belonging to a specialised health workforce. These benefits reflect the current over-representation of Māori and Pacific peoples in the lowest-skilled occupational groups (or in skill groups with low future demand) and under-representation in the more highly skilled occupational groups [20,25,26]. In the NZ context, development of an indigenous health workforce also reflects a commitment to the indigenous rights of Māori as *tangata whenua*^*a*^ [27]. Therefore, overcoming Māori health workforce inequities reflects an indigenous rights imperative [28,29].

The shortage of indigenous and ethnic minority health professionals is related to a complex mix of historical, political, demographic, cultural, academic and financial factors [30]. Worldwide, higher education institutions attempt to support indigenous and ethnic minority health workforce development via the provision of equity-targeted admission policies, alternative bridging/foundation pathways and comprehensive support programmes [17,18,20,30-32]. These activities are especially important when there are differences in secondary school education outcomes by ethnicity, as is the case in NZ. For example, in 2009 the proportion of school leavers who met the requirements for University Entrance (UE) was 27% for Māori and 33% for Pacific compared to 54% for Pākehā^b^ [33,34].

With a view to addressing health inequities, this article describes research into how to select health professional students via a NZ tertiary admission pathway. Selection tools used by universities include a mix of methods aimed at identifying both ‘cognitive’ variables (i.e. measures of academic merit or readiness) and ‘non-cognitive’ variables (i.e. measures of personal characteristics or qualities) necessary for success within health professional programmes and ultimately as health practitioners [35,36]. Common selection tools have included secondary school results, undergraduate Grade Point Average (GPA), standardised aptitude tests (e.g. UMAT), college admission tests (e.g. MCAT) and interview-related techniques (e.g. panel interview, Multiple Mini Interview (MMI)) [13].

Understanding is required of how these tools operate within an equity-targeted admissions context. How can indigenous and ethnic minority applicants be best assessed for their potential to ‘succeed’ in what can often be highly competitive and demanding higher education programmes immersed within a European-centric institution? [37-42]. How do universities determine whether indigenous and ethnic minority students require additional educational and transitioning support via alternative routes such as bridging foundation programmes? This is particularly relevant for many indigenous and ethnic minority applicants given their greater risk of socio-economic disadvantage that is associated with poorer outcomes in the first year of tertiary study [2,43] and lower likelihood of inter-generational tertiary experience that universities draw on to help students navigate and transition into the academy [40,44].

### Institutional context

The Faculty of Medical and Health Sciences at the UoA provides an equity-targeted admissions process for applicants with Māori and Pacific ancestry who wish to enter health professional programmes via MAPAS [27,45,46]. In operation since 1972, with an original focus on medical admission, MAPAS now includes nursing, health sciences and pharmacy (with optometry joining the Faculty in 2013). In the 1990s, a high-level equity strategy was adopted by the FMHS known as *Vision 20:20* that articulated an aim for all health professions in the NZ health workforce, particularly frontline clinical roles, to be made up of at least 10% Māori and Pacific peoples by the year 2020. Additional programmes were introduced alongside MAPAS to contribute to the *Vision 20:20* initiative including a one year bridging/foundation programme for Māori and Pacific students in 1999 *Hikitia Te Ora- Certificate in Health Science (CertHSc)* and a Māori recruitment programme - *Whakapiki Ake Project (WAP)* in 2003. Since their implementation, the number of Māori and Pacific students entering the faculty has increased (e.g. Māori and Pacific students now make up approximately 25% of the yearly medical intake) and student performance in terms of pass rates and completion rates have also improved over time [27].

### MAPAS Admissions process

Student selection under MAPAS was historically based around an interview between applicants (and their family) and a panel of Māori and Pacific academic, health sector and community representatives. Applicants were required to show evidence of active participation in and involvement with Māori and or Pacific culture as well as adequate secondary school or tertiary results [45]. In 2006, the MAPAS admissions process underwent significant change due to broad concerns with the academic performance of MAPAS students, the need to develop better entry criteria for the CertHSc programme and a desire to avoid value-laden cultural judgments associated with the original selection process [27].

The current MAPAS General Interview (‘interview’) aims to identify an applicant’s intended health career choice (which are often multiple) and determine the *best starting point* for them to succeed in achieving their career aspirations. In order to receive an interview offer, applicants must provide certified evidence of their indigenous Māori and/or Pacific ancestry prior to an interview offer being made. The interview requires applicants to attend one whole day in December held approximately three months before the New Zealand tertiary year commences. The interview is delivered within an indigenous cultural context where the day begins with a traditional ceremony of welcome known as the *pōwhiri* and accompanying *whānau* (families) are hosted for the duration of the day whilst the applicants undergo testing. Whānau-targeted activities including campus tours and interactive information seminars are provided to inform families of the interview process and improve understanding of the transitioning challenges associated with tertiary study.

The interview consists of a MMI, a mathematics test to assess basic numeracy skills and an English test to assess basic literacy skills. The mathematics and English tests are marked for two contexts: 1) foundation/bridging level study (i.e. pass/borderline/fail) and 2) degree level study (i.e. pass/borderline/fail). The MMI includes four 8-minute stations assessing *career aspirations (e.g. career intentions and knowledge of study pathways)*; *academic preparation (e.g. exposure to prerequisite subjects, prior tertiary qualifications)*; *family support (e.g. available family support for student during study)* and *student information (e.g. work, sport, religious commitments, living arrangements, financial support)*. Similar to the objective testing, two levels of subjective assessment are made at each MMI station: 1) the potential to succeed within the CertHSc (i.e. *few concerns*, *some concerns*, or *major concerns*) and 2) the potential to succeed within a degree-level programme (i.e. *few concerns*, *some concerns*, or *major concerns*). Informed by relevant literature [47], the MMI stations were designed by FMHS academic and MAPAS professional staff knowledgeable of common factors associated with MAPAS student academic success and failure [48].

Data from MAPAS testing are collated, assessed by senior *Vision 20:20* academic and professional staff and a provisional MAPAS December recommendation is made. Each applicant and their family members are offered an individualised feedback session on the day of their interview to explain their results and provisional recommendation. The MAPAS December recommendation is provisional because secondary school results in NZ are not available until early January [48]. An online multi-media vignette has been developed by *Vision 20:20* to assist applicants and their whānau to better understand the interview process (to view see: https://www.fmhs.auckland.ac.nz/en/faculty/tkhm/vision-20-20/m_ori-and-pacific-admission-scheme.html).

The NZ secondary school qualification system known as *The National Certificate in Educational Achievement (NCEA)* provides the majority of secondary school results for MAPAS interviewees [48]. Secondary school students gain credits for each NCEA subject studied and each credit is also awarded at an Achieved, Merit or Excellence level. The top 80 credits (representing the bulk of all credits achieved) in the five best subjects are weighted based on the level of achievement attained and these scores are combined to calculate an overall ‘rank score’ that acts as an overall indicator of the students’ academic performance [49]. Guaranteed entry into FMHS degree-level programmes takes account of both rank score and number of credits achieved for preferred subjects i.e. Table A (language-rich) and Table B (science/mathematics-rich) subjects (Table 1) [50]. Once school results are released in January, the final MAPAS recommendation is confirmed using a combination of the interview and secondary school results. The final MAPAS recommendation is communicated to applicants via a written letter with individualised explanations for the recommendation being made.

**Table 1:** **Table A and B secondary school subjects for University of Auckland entry**

**Table A**	**Table B**
Classical studies	Accounting
English	Biology
Geography	Chemistry
History	Economics
History of Art	Mathematics
Te Reo Māori OR	Physics
Te Reo Rangatira	

MAPAS considers three starting point options: (1) ‘Bachelor’ i.e. direct entry into a FMHS undergraduate programme; (2) ‘CertHSc’ i.e. an additional year of bridging/foundation study, and (3) ‘Not FMHS’ i.e. students are advised to study in a programme outside of the FMHS which often indicates their need to develop foundational science, mathematics or English academic knowledge. Ideally, CertHSc students will have had exposure to the equivalent of at least two Level 3 NCEA science subjects (e.g. biology, chemistry or physics), and be able to demonstrate academic and pastoral potential for success within this demanding bridging/foundation programme that is restricted to approximately 70 students [48]. The final MAPAS recommendation does not override the guaranteed entry criteria set for a FMHS degree programme (based on rank score and Table A/B requirements). Therefore, if a MAPAS interviewee receives a degree-level programme offer that differs from the advice provided by MAPAS (e.g. to start at the bridging/foundation level) they retain control over whether they accept the programme offer or follow MAPAS advice for their best starting point.

In summary, the MAPAS admissions process aims to recommend best starting points for academic success based on a broad mixture of factors including: the proximity of a MAPAS applicant to guaranteed entry criteria (reflecting secondary school subject exposure and achievement); the potential for success in the first year of tertiary study; the academic requirements of any intended health professional programme including medicine that commences in Year 2 (dependent on academic performance in the first year of tertiary study) and socio-cultural factors that are likely to impact on academic success. The combination of both cognitive (i.e. objective) and non-cognitive (i.e. subjective) selection tools guides the final MAPAS recommendation resulting in an intended holistic assessment of academic potential for success.

### Research aim

This research project aims to describe the current MAPAS admissions process using quantitative data and explore the predictive effect of admission variables on MAPAS recommendations.

### Research objectives

The specific objectives of this research are:

- Objective 1: To describe the MAPAS applicant cohort using secondary data from 2008 to 2012.
- Objective 2: To describe the quantitative admission variables available to MAPAS associated with the new admissions process.
- Objective 3: To identify the predictive effect of admission variables on the final MAPAS recommendation made in January next year.

## Methods

### Methodology

This study utilised a Kaupapa Māori Research (KMR) approach, broadly defined and inclusive of Pacific research methodologies [51,52]. KMR is based on a number of key principles relevant to research for Māori and Pacific students. In this instance it aims to provide: a commitment to ensuring that the research outputs will have positive benefits for Māori and Pacific students and communities; an explicit challenge to reject ‘victim blame’ and ‘cultural deficit’ analyses when interpreting data [53]; promoting a structural analysis that rejects findings that suggest the culture of Māori or Pacific students are to blame for their educational failure and ensuring that any recommendations made from the research aim to facilitate Māori and Pacific student success. Recent research supports the notion of ‘success’ as including academic achievement alongside accomplishment of personally significant goals including the development of cultural skills or knowledge within a tertiary setting [37,38].

A broad KMR approach has been taken for this research involving both Māori and Pacific students as the issues associated with power, privilege and agency within society are hypothesised to act similarly on both groups [28,29]. A formal advisory group made up of Māori, Pacific, faculty and health programme representatives with research and academic expertise was regularly convened to oversee the research design and data analysis via a process of active dialogue and debate across disciplines. This approach allowed for senior Māori and Pacific oversight of the research and is consistent with KMR and Pacific research methodological principles (e.g. the *Talanoa* process^c^) [54].

### Data sources

Data were obtained from the MAPAS admissions database and the UoA’s centralised student data management system *Student Services Online (SSO)* for all MAPAS applicants interviewed from 2008 to 2012. Ethical approval for the study was granted by the University of Auckland Human Participants Ethics Committee (Ref 8110). In alignment with ethics protocols that ensured student confidentiality, raw data from multiple datasets were combined, duplicates removed, and data de-identified by a research assistant prior to analysis.

### Data variables

Descriptive data available at the time of application includes: MAPAS General Interview Year (2008–2012); Gender (Female, Male); Ancestry (Māori, Pacific, Both) and Admission Category (School Leaver, Alternative Admission^d^) (Table 2).

**Table 2:** **Descriptive summary of MAPAS interview attendees’ demographics and outcome variables, 2008–2012**

**Descriptive variables**	**MAPAS IV attendees 2008 – 2012**			
	**Māori**	**Pacific**	**Both**	**Total (%)** ^**#**^
	***n (%)***	***n (%)***	***n (%)*** ^***+***^	***n (%)***
Total Cohort	319 (35%)	530 (58%)	68 (7%)	918
MAPAS IV year				
2008	42	59	8	109 (12)
2009	55	89	6	150 (16)
2010	73	151	15	239 (26)
2011	72	123	20	215 (24)
2012	77	108	19	205 (22)
Gender				
Female	223 (70)	391 (74)	48 (71)	662 (72)
Male	96 (30)	139 (26)	20 (29)	256 (28)
Admit category				
SL*	222 (70)	390 (74)	41 (60)	653 (71)
AA*	97 (30)	140 (26)	27 (40)	265 (29)
Final Jan Rec.				
Certificate	154 (48)	251 (47)	22 (32)	428 (47)
Bachelors	541 (17)	53 (10)	10 (15)	117 (13)
Not FMHS*	89 (28)	208 (39)	35 (51)	332 (36)
Missing	22 (7)	18 (4)	1 (1)	41 (4)

*SL = School Leaver, AA = Alternative Admission, FMHS = Faculty of Medical and Health Sciences

^#^Note that one student with missing Ancestry has been included in the Totals.

^+^Proportion calculations may not reflect 100% due to rounding issues.

Admissions process data available following the interview in December includes: MAPAS Mathematics test results (%); MAPAS English test results (%); MMI station results as Some or Major Concerns (SMC) versus Few Concerns (FC); Overall Assessment for CertHSc or Bachelor entry (SMC, FC); and provisional December Recommendation (CertHSc, Bachelor, Not FMHS) (Table 3).

**Table 3:** **Admission process variables (December and January) for MAPAS interview attendees (2008–2012)**

**MAPAS admissions process variables**	**MAPAS IV attendees 2008 – 2012**			
	**Māori (n = 319)**	**Pacific (n = 530)**	**Both (n = 68)**	**Total (n = 918)**
***Continuous variables***	***Mean ± SD***	***Mean ± SD***	***Mean ± SD***	***Mean ± SD***
MAPAS Maths test	75.2 ± 20.5	70.3 ± 22.1	68.9 ± 22.2	71.9 ± 21.7
MAPAS English test	67.6 ± 14.5	61.2 ± 15.9	67.0 ± 14.1	63.9 ± 15.6
NCEA School results				
Rank Score°	185.0 ± 65.2	157.3 ± 61.7	162.1 ± 71.8	166.8 ± 64.8
L3 English	15.6 ± 6.9	14.4 ± 6.5	13.9 ± 7.5	14.8 ± 6.7
L3 Biology	15.4 ± 6.7	13.0 ± 6.3	12.2 ± 6.3	13.7 ± 6.5
L3 Chemistry	13.6 ± 7.8	11.8 ± 7.3	14.7 ± 8.9	12.6 ± 7.6
L3 Physics	15.7 ± 7.8	13.1 ± 7.9	15.3 ± 8.7	14.1 ± 7.9
L3 Maths	22.2 ± 13.4	20.6 ± 12.7	20.3 ± 11.4	21.1 ± 12.9
*Categorical variables*	*n*	*n*	*n*	*n (%)*
MMI Cert level*				
Whānau Support				
FC	238	377	48	663 (72)
SMC	80	153	19	253 (28)
Missing	1	0	1	2 (0)
Academic Prep.				
FC	227	325	39	591 (64)
SMC	91	205	28	325 (35)
Missing	1	0	1	2 (0)
Career Aspirations				
FC	237	362	42	642 (70)
SMC	81	168	25	274 (30)
Missing	1	0	1	2 (0)
Student Information				
FC	237	341	46	625 (68)
SMC	81	189	20	290 (32)
Missing	1	0	2	3 (0)
MMI Bachelor level*				
Whānau Support				
FC	194	276	40	510 (56)
SMC	124	254	27	406 (44)
Missing	1	0	1	2 (0)
Academic Prep.				
FC	131	176	20	327 (36)
SMC	186	353	46	586 (64)
Missing	2	1	2	5 (0)
Career Aspirations				
FC	117	166	20	304 (33)
SMC	201	364	47	612 (67)
Missing	1	0	1	2 (0)
Student Information				
FC	151	221	36	409 (45)
SMC	167	308	30	505 (55)
Missing	1	1	2	4 (0)
Overall SMC @ Cert				
Yes	162	350	42	555 (60)
No	156	177	25	358 (39)
Missing	1	3	1	5 (0)
Overall SMC @ Bach				
Yes	301	506	60	868 (95)
No	17	22	7	46 (5)
Missing	1	2	1	4 (0)
Dec Rec. (M = 24)				
Certificate	143	244	22	410 (46)
Bachelors	94	103	13	210 (23)
NOT FMHS	74	169	31	274 (31)
Missing	8	14	2	24 (3)
Any 2 sciences^				
Yes	157	258	23	439 (48)
No	75	150	22	247 (27)
Missing	87	122	23	232 (25)

*FC = Few Concerns, SMC = Some or Major Concerns; °Rank Score and L3 subject results analysis was completed for applicants who completed NCEA only. Excludes CIE, IB, international students, Alternative Admission applicants and missing data; ^Any 2 sciences was calculated for all applicants who had available subject results for any two of the three applied science subjects (physics, biology, chemistry) and includes NCEA, CIE and IB. The ‘missing’ numbers therefore represent applicants who did not have school results available (i.e. Alternative Admission applicants).

January data variables include: NCEA rank score (out of 320), Level 3 NCEA subject credits (number of credits in English, biology, chemistry, physics, mathematics) for all applicants who had completed NCEA; exposure to any 2 sciences of biology, chemistry or physics (yes, no) for all applicants who had completed NCEA, CIE or IB^e^ at some time (includes Alternative Admission applicants); and Final MAPAS recommendation (CertHSc, Bachelor, Not FMHS) (Table 3).

### Data analysis

All data were recorded in a Microsoft Office Excel spread sheet and imported to SAS version 9.3 (SAS Institute, Cary, NC, USA) for further analysis. Descriptive summaries were provided for all MAPAS interviewees (2008–2012) by ancestry and overall. Continuous variables were presented as mean and standard deviation (SD). Categorical variables were presented as frequencies (n) and percentages (%). Missing data were reported in the tables.

The agreement between the December (provisional) and January (final) recommendations were tested using the Kappa statistic with associated 95% confidence interval (CI) and p-value. Multiple logistic regression models were used to estimate the predicted effects of the December interview process on January recommendations (CertHSc, Bachelors, Not FMHS). Three confounding variables were pre-defined and adjusted for in all models: MAPAS interview year, gender and ancestry. For the purpose of analysis, those with both Māori and Pacific ancestry were weighted twice i.e. counted along with both those with the ancestry indicated as Māori, and those with ancestry indicated as Pacific. The adjusted odds ratio (OR) and its 95% confidence interval were estimated for all predictors of interest. For categorical variables, this indicates the odds of being recommended for a *best starting point* relative to the odds of being recommended for that same *starting point* if the interviewee falls in the reference category (indicated with an OR of 1.00). For continuous variables, this indicates the odds of being recommended for a *best starting point* if the interviewee’s predicted score increases by one unit, relative to the odds at the reference value. An OR of 1 suggests no difference, i.e. the null hypothesis. Alternatively, an OR greater/less than 1 suggests a higher/lower chance (in terms of odds) of being recommended for a *best starting point*. All statistical tests were two-sided at 5% significance level. Missing data were not imputed, and there was no adjustment for multiple comparisons.

## Results

### Descriptive variables

A total of 918 Māori and Pacific applicants completed the MAPAS admissions process between 2008 and 2012 (Table 2). The number of interviewees per year has doubled over the five-year period with 205 interviews being conducted in 2012 (for 2013 entry). Of those interviewed under MAPAS, 319 (35%) were Māori, 530 (58%) were Pacific, and 68 (7%) identified ancestry as both Māori and Pacific. Similar proportions of Māori, Pacific and those students with both Māori and Pacific ancestry were interviewed over the five-year period reflecting an absolute increase in the numbers of MAPAS interviewees with little change in ancestry distribution. Approximately two thirds of interviewees were female (662, 72%). The majority of the MAPAS interviewees applied directly from secondary school (653, 71%) with just over one quarter of interviewees from alternative pathways or non-school leaver entry i.e. Alternative Admission (265, 29%). The proportion of Alternative Admission interviewees was slightly higher for Pacific versus Māori students (36% versus 30% respectively).

### Admission process variables

The mean percentage mark for the MAPAS mathematics test was 71.9% with a standard deviation of 21.7%. The mean percentage mark for the MAPAS English test was 64.0% with a standard deviation of 15.6%. This represents a *fail* for Bachelor-level study and a *pass* for CertHSc-level study as the best starting point of entry across both assessments. Pacific interviewees had slightly lower average marks on both the MAPAS mathematics and English testing compared to Māori (70.3% vs 75.2% and 61.2% vs. 67.6% respectively).

Approximately two thirds of all interviewees were assessed as having *few concerns* for CertHSc-level entry at each MMI station (64-72%), compared to approximately one third of all interviewees assessed as having *some* or *major concerns* (28-35%). A different pattern was observed for Bachelor-level entry with approximately half to two thirds of all interviewees being assessed as having *some* or *major concerns* (44-67%) and approximately one third to half of all interviewees assessed as having *few concerns* for Bachelor-level entry at each MMI station (33-56%).

The overall MAPAS assessment (reflecting the combination of results from the mathematics, English and MMI testing) identified the majority of MAPAS interviewees (95%) as having *some* or major concerns for Bachelor-level study. In addition, nearly two thirds of all MAPAS interviewees (60%) were identified as having *some* or *major concerns* for CertHSc-level study following overall MAPAS assessment. This proportion was higher for Pacific (66%) compared to Māori (51%).

Nearly half of the MAPAS interviewees (46%) received a December recommendation for CertHSc as their best starting point, followed by Not FMHS (31%) and Bachelor (23%). The average rank score (out of a total of 320) achieved by MAPAS interviewees applying between 2008 and 2012^f^ was 167 ± 65^g^ with Māori interviewees having a higher average rank score (185 ± 65) compared to Pacific (157 ± 62). Seventy six percent of all MAPAS interviewees taking NCEA Level 3 subjects had exposure to English, biology and mathematics, two thirds to chemistry and one third to physics. The average number of credits achieved by all MAPAS interviewees were 12.6 ± 7.6 for chemistry and 14.8 ± 6.7 for English; approximately 1–5 credits below requirements for guaranteed entry within FMHS Bachelor level programmes (i.e. 16–18 credits for Table A or B subjects). The average credits achieved by Pacific interviewees were consistently lower than Māori interviewees across all subjects. Twenty-seven percent of all MAPAS interview attendees did not have exposure to any 2 sciences at Level 3 (Table 3).

Of the total 918 MAPAS applicants interviewed, less than half received a final January recommendation to start at the CertHSc (428, 47%). Only 117 (13%) received a final MAPAS recommendation to start tertiary study at the Bachelor level and 38% (332) received a final MAPAS recommendation to start tertiary study outside of the FMHS (potentially including other UoA faculties or other tertiary institutions) (Table 2)^h^. Although a greater proportion of applicants interviewed by MAPAS were Pacific compared to Māori over the study time period, a similar proportion was recommended to start at the CertHSc in January (48% and 47% respectively). Seventeen percent of Māori applicants were recommended to start study at the Bachelor level compared to 10% for Pacific, with a greater proportion of Pacific applicants being recommended to start their journey outside of the FMHS following receipt of school results (39% versus 28% for Māori) (Table 2).

### Predictive effect of admission variables on MAPAS recommendations

Table 4 presents the predicted effects of MAPAS admission variables on the final January recommendation. Strong associations with a recommendation of Not FMHS (i.e. a pathway outside of the FMHS) were observed for MMI results (SMC versus FC) at Bachelor level for *academic preparation* (OR^i^:4.992, CI^j^:3.3-7.5; p < 0.0001) and *career aspirations* stations (OR:2.739, CI:1.9-3.9; p < 0.0001). A lower performance on the MAPAS mathematics (OR:0.957, CI:0.949-0.965) and English tests (OR:0.972, CI:0.961-0.984); lower rank score (OR:0.968, CI:0.963-0.973) and not having exposure to any 2 sciences (OR:0.84, CI:0.057-0.125) predicted a higher chance of receiving a recommendation of Not FMHS (all p-values <0.0001). Having some or major concerns within *Whānau support* was strongly associated with a Not FMHS recommendation at the Certificate level in particular (OR:1.867, CI:1.341-2.598).

**Table 4:** Predicted effects* on January recommendations for MAPAS interviewees 2008 – 2012 (n = 918)

**Regression analysis results**	**January recommendation**					
	**CertHSc**		**Bachelors**		**NOT FMHS**	
***Predictors***	***Odds ratio (95% CI)***	***P value***	***Odds ratio (95% CI)***	***P value***	***Odds ratio (95% CI)***	***P value***
MAPAS Maths test	**1.023 (1.016, 1.031)**	<0.0001	**1.043 (1.028, 1.059)**	<0.0001	**0.957 (0.949, 0.965)**	<0.0001
MAPAS English test	1.010 (1.000, 1.020)	0.0583	**1.042 (1.023, 1.061)**	<0.0001	**0.972 (0.961, 0.984)**	<0.0001
MMI for Certificate level						
Whānau Support						
Few concerns	1.00		1.00		1.00	
Some/Major concerns	0.792 (0.582, 1.078)	0.1387	**0.392 (0.214, 0.719)**	0.0025	**1.867 (1.341, 2.598)**	0.0002
Academic preparation						
Few concerns	1.00		1.00		1.00	
Some/Major concerns	**0.460 (0.342, 0.620)**	<0.0001	**0.200 (0.101, 0.395)**	<0.0001	**3.931 (2.882, 5.361)**	<0.0001
Career aspirations						
Few concerns	1.00		1.00		1.00	
Some/Major concerns	**0.731 (0.538, 0.994)**	0.0457	**0.296 (0.165, 0.562)**	0.0002	**2.236 (1.614, 3.097)**	<0.0001
Student Information						
Few concerns	1.00		1.00		1.00	
Some/Major concerns	0.860 (0.634, 1.165)	0.3298	0.974 (0.583, 1.630)	0.9211	1.208 (0.872, 1.673)	0.2566
MMI for Bachelor level						
Whānau Support						
Few concerns	1.00		1.00		1.00	
Some/Major concerns	0.838 (0.629, 1.117)	0.2274	0.676 (0.412, 1.109)	0.1208	**1.498 (1.088, 2.062)**	0.0133
Academic preparation						
Few concerns	1.00		1.00		1.00	
Some/Major concerns	**0.669 (0.489, 0.915)**	0.0118	**0.212 (0.129, 0.346)**	<0.0001	**4.992 (3.343, 7.456)**	<0.0001
Career aspirations						
Few concerns	1.00		1.00		1.00	
Some/Major concerns	0.860 (0.638, 1.160)	0.3229	**0.328 (0.209, 0.513)**	<0.0001	**2.739 (1.906, 3.937)**	<0.0001
Student Information						
Few concerns	1.00		1.00		1.00	
Some/Major concerns	0.840 (0.638, 1.105)	0.2132	0.692 (0.443, 1.081)	0.1060	**1.508 (1.104, 2.059)**	0.0098
NCEA Rank Score	**1.008 (1.006, 1.011)**	<0.0001	**1.043 (1.034, 1.052)**	<0.0001	**0.968 (0.963, 0.973)**	<0.0001
NCEA Level 3 Subjects						
English	**0.878 (0.804, 0.960)**	0.0041	**1.519 (1.234, 1.871)**	<0.0001	0.886 (0.752, 1.042)	0.1427
Biology	1.011 (0.929, 1.100)	0.8028	**1.154 (1.006, 1.323)**	0.0404	0.849 (0.702, 1.026)	0.0907
Chemistry	**0.919 (0.847, 0.997)**	0.0420	**1.150 (1.003, 1.318)**	0.0450	1.145 (0.943, 1.390)	0.1707
Physics	1.012 (0.947, 1.081)	0.7280	1.038 (0.940, 1.146)	0.4667	0.887 (0.746, 1.056)	0.1784
Maths	1.032 (0.988, 1.078)	0.1597	0.980 (0.919, 1.044)	0.5242	**0.879 (0.783, 0.987)**	0.0296
Any 2 Sciences?						
No	1.00		1.00		1.00	
Yes	**3.777 (2.730, 5.227)**	<0.0001	**7.897 (3.855, 16.175)**	<0.0001	**0.084 (0.057, 0.125)**	<0.0001

Logistic Regression adjusted for MAPAS interview year, Gender and Ancestry. An OR of 1 suggests no difference, i.e. the null hypothesis. Bold font indicates statistically significant results (P < 0.05).

Some examples on the interpretation of OR: An increase in MAPAS Maths test result corresponds to increased odds of getting a CertHSc recommendation in January, by a factor of 1.023 for every percentage point increase in Maths result. Having some/major concerns for Whanau Support at CertHSc level in MMI corresponds to decreased odds of getting a CertHSc recommendation in January, by a factor of 0.792 compared to few concerns.

The significant predictors associated with a final recommendation of Bachelor included: exposure to any 2 Sciences (OR:7.897, CI:3.855-16.175; p < 0.0001); higher scores on the MAPAS mathematics test (OR:1.043, CI:1.028-1.059; p < 0.0001), MAPAS English test (OR:1.042, CI:1.023-1.061; p < 0.0001), and better secondary school outcomes including rank score (OR:1.043, CI:1.034-1.052; p < 0.0001); NCEA Level 3 English credits (OR:1.519, CI:1.234-1.871; p < 0.0001); biology credits (OR:1.154, CI:1.006-1.0323; p < 0.0404) and chemistry credits (OR:1.150, CI:1.003-1.318; p < 0.0450).

Applicants who were assessed as having some or major concerns within the MMI stations of *academic preparation* (OR:0.212, CI:0.129-0.346; p < 0.0001) and *career aspirations* (OR: 0.328, CI:0.209-0.513; p < 0.0001) were less likely to receive a Bachelor recommendation compared to those who were assessed as having few concerns. Significant associations were found between the following predictor variables and a final CertHSc recommendation: MMI *academic preparation* at both Bachelor (OR: 0.669, CI:0.489-0.915; p = 0.0118) and CertHSc level (OR: 0.460, CI:0.342-0.620; p < 0.0001); any 2 Sciences (OR: 3.777, CI:2.730-5.227; p < 0.0001); MAPAS mathematics test result (OR: 1.023, CI:1.016-1.031; p < 0.0001); NCEA rank score (OR: 1.008, CI:1.006-1.011; p < 0.0001); English (OR: 0.878, CI:0.804-0.960; p = 0.0041) and chemistry subject credits (OR: 0.919, CI:0.847-0.997; p = 0.0420).

It is worth noting that a good level of agreement was found between December and January recommendations for CertHSc, Bachelors, and Not FMHS (Kappa Coefficient 0.46, CI: 0.415-0.505; p < .0001).

## Discussion

Our findings raise concerns about the ability of the secondary education sector to prepare Māori and Pacific students adequately for tertiary health professional study. The average rank score of MAPAS interviewees was approximately 40–80 credits below the guaranteed rank score for FMHS bachelor programme offers made during this time period (167 versus 210–250 respectively). Despite the majority of MAPAS applicants having exposure to important subjects for health study i.e. English, biology, chemistry, physics and mathematics, the mean number of credits achieved within these subjects were approximately 1–2 credits below guaranteed entry for nursing and 3–5 credits below guaranteed entry for health sciences and pharmacy programme offers. Of concern, MAPAS applicants had limited exposure to multiple science subjects in their final year of secondary study despite this being recommended for success within tertiary health programmes [26,50,55].

Our findings are consistent with the increasing evidence of differential secondary school outcomes by ethnicity in NZ, particularly within science subjects necessary for health careers [33,34,56]. For example, in 2012 there were 7,493 Māori and 32,361 non-Māori students aged 17 years enrolled at secondary school. Of these, only 16.4% of Māori versus 35.6% of non-Māori students participated in a science subject and only 8.2% of Māori attained more than 14 credits in one science subject compared to 24.8% of non-Māori [56]. Available data show consistent evidence of Pacific students being less likely to receive an NCEA Level 2 or University Entrance qualification on leaving secondary school compared to non-Pacific students [3,33]. Our findings show that although Pacific applicants are more numerous than Māori applicants, they have been less well prepared for health professional study than Māori applicants and are less likely to be recommended to begin their journey within the FMHS programmes.

We note that we cannot clarify whether MAPAS applicants are representative of the national cohort of Māori and Pacific or total school leavers with respect to rank score as this information is not reported nationally. Given that rank score is increasingly being used by tertiary institutions to determine entry into specialised programmes of study we recommend that government agencies record and track the association between rank score and tertiary entry by ethnicity in the future. Regardless, this evidence suggests that New Zealand secondary schools are (a) failing to enrol sufficient numbers of Māori and Pacific students in subjects relevant to health professional study, (b) failing to retain sufficient numbers of Māori and Pacific students until their final year of study and (3) failing to achieve parity in educational outcomes for those Māori and Pacific students who do stay compared to non-Māori non-Pacific students.

Similar inequities in educational outcomes are seen internationally [57-60]. Calls have been made for secondary educational institutions to provide indigenous and ethnic minority students with classroom and learning opportunities that will enhance achievement within science subjects in particular [60]. The potential inability of educators, and the education system in which they are operating, to provide culturally-safe and effective teaching and learning contexts for indigenous and ethnic minority secondary school students should be addressed. Bishop, Berryman, Tiakiwai and Richardson [61] found that deficit theorising by teachers was the major impediment to Māori educational achievement. They recommend the provision of professional development for educators utilising non-confrontational situations to foster personal critical self-reflection in order to improve classroom interactions for indigenous students.

Our findings (and admission experience) support the need to explore the contribution of inadequate careers advice in the context of health professional study [30,44,57,62]. Chesters et al. [62] conclude that secondary school advisors lack the knowledge base required to support indigenous students into health careers. Re-enforcement of negative stereotypes, either intentionally or unintentionally, by careers advisors and secondary school teachers may be contributing to our findings of inadequate subject choices [57]. McKinley and Madjar [44] note that to keep students on an academic pathway requires careful goal setting, regular monitoring, academic counselling and family engagement. Their findings highlight stumbling blocks for successful tertiary transition as including *“the lack of adequate academic preparation and guidance at school, absence of adequate mentors, lack of clear goals and failure to be challenged to their potential rather than settle for the minimum needed to pass”* (p. 250).

Ironically, the recent growth in health workforce recruitment (e.g. health academies, science camps, work-based exposure programmes) targeting Māori and Pacific youth may be contributing to our findings of poor tertiary preparation [6,22,63-66]. Focused on promoting health career aspirations, many of these programmes sit outside of the secondary and/or tertiary education sectors. We suspect that this may have resulted in an increase in the number of applicants to tertiary health programmes (associated with an increase in health career aspirations). However, there has been a lack of focus on ensuring that these applicants are equipped with the necessary academic prerequisites (exposure to science subjects and sufficient NCEA credits achieved) to gain entry into and success through health professional programmes. Therefore, recruitment programmes should achieve both outcomes of health recruitment i.e. increasing aspiration *and* successful academic preparation for higher education pathways, particularly for Pacific students. Further investigation is required. A comprehensive and integrated pipeline model of recruitment that operates across secondary and tertiary education sectors to provide early exposure, transitioning, retention/completion and post-graduate support is recommended [30]. Greater inclusion of indigenous and ethnic minority families and communities into the educational experiences and career choices of their children is also advised [38,39,60,67-69]. It is important to note that the MAPAS admission process is one of many Māori and Pacific health workforce development initiatives that operate across this recruitment pipeline. In order to achieve health equity, additional interventions focused on multiple levels within society are required including the elimination of discrimination within secondary education, healthcare delivery, socio-economic determinants of health and the broader socio-political environment.

To our knowledge, this is the first study that has explored the application of the MMI and objective testing results within an equity-targeted and indigenous cultural context. Overall the cognitive and non-cognitive selection methods used in the MAPAS admission process are consistent. For example in December, approximately two-thirds of the MAPAS applicants were assessed as having some or major concerns at MMI and objective testing for entry into Bachelor-level study. These trends continue in January, with secondary school results reinforcing the assessment that the majority of MAPAS interviewees are not ‘university-ready’ for health professional programmes offered by the UoA.

Strong associations were seen between the predictor variables of exposure to Any 2 Sciences, secondary school rank score and the MAPAS maths test and all three final January recommendations made by MAPAS i.e. Bachelor, CertHSc or Not FMHS. Individual MMI stations had mixed associations with the a*cademic preparation* and *careers aspirations* stations more consistently associated with the January outcome variables. The finding of mixed associations via individual MMI station is not surprising, given that the MMI method is designed to reflect the combination of individual marker assessments [35]. A total, combined assessment that draws from the combination of all MMI stations to overcome context specificity (alongside objective testing) is the intended outcome of the MAPAS MMI process and our findings support the continuation of this approach [47,70].

Our results reinforce the need to utilise multiple selection tools, above and beyond academic secondary school results, to assist in the selection process. This may reflect the MAPAS cohort mix, with one third of all applicants being Alternative Admission alongside a school leaver population with secondary school outcomes that are generally inconsistent with tertiary entry requirements for health professional study. This context is likely to be common to other indigenous and ethnic minority groups experiencing differential outcomes from secondary education [71]. Therefore, the current MAPAS admissions process may provide an exemplar for other tertiary institutions looking to widen participation via admission processes that recognise students as individuals with unique circumstances [72].

The MAPAS approach of identifying the *best starting point* for entry towards a health career can clash with the institutional information provided to applicants regarding guaranteed programme entry. The potential ‘mismatch’ between FMHS programme offers and MAPAS recommendations reflect the different intent of the two admission processes. Driven by funding caps imposed on New Zealand universities by Government, programme entry criteria must maintain a level of flexibility to ensure that enough students can enter (but not too many) [17,73].

Within this context, a minimum rank score and a minimum number of Table A or B subject credits are set with the promise of guaranteed entry if achieved by any applicant. However, the list of subjects included in each table remains broad in nature [50]. This allows applicants with no science exposure to enter science-heavy undergraduate programmes simply because they have met the minimum requirements for guaranteed entry. Building on previous research, we hypothesise that this approach is not helpful when applied to a cohort of applicants who experience multiple barriers for successful transitioning into university study [44].

Our findings support the ongoing delivery of the CertHSc bridging/foundation programme given the small proportion of MAPAS applicants who were assessed as being ready for direct Bachelor-level entry (only 13% of the total cohort). Universities committed to growing an indigenous and ethnic minority health workforce are likely to need alternative pathways if the majority of their applicants face educational and social barriers that limit direct entry into tertiary health study [32]. Aiming for a comprehensive suite of interventions is encouraged including the use of admission/quota policies, tertiary enrichment programmes and the provision of pre-matriculation or application support within a pipeline approach to recruitment [30].

### Limitations

Age was not specifically recorded by MAPAS during the study period, however the effect is likely to be minimal given that the majority of MAPAS applicants were school leavers and therefore close in age range. Some variables (particularly exposure to any 2 Sciences, December Recommendation and January Recommendation) had missing data ranging from 3-25% due to information being unrecorded or unavailable. It is reassuring that strong associations were still identified for these variables despite the missing data.

In this study we have considered a total MAPAS cohort that combined both Māori and Pacific interviewees in the data analysis, although ancestry was controlled in all models. No major differences were found when data analyses were conducted on sub-groups disaggregated by ancestry providing statistical support for presenting combined ancestry data. We acknowledge that this may not be ideal from an indigenous rights or Pacific-centric perspective as it may be interpreted as assuming Māori and Pacific ancestry are homogenous. However, combining data supports the explicit critique of ‘society’ on students with Māori and Pacific ancestry; rather than Māori and Pacific ‘ancestry’ itself. This positioning aligns with recent calls to ensure that Kaupapa Māori approaches reflect critical theory and structural analyses and avoid *“domestication”* via a focus on cultural elements only [74]. The articulation of Pacific-methodological approaches [51] both juxtaposed to, and separate from, Kaupapa Māori research is recommended within future research to advance the development of both approaches within a New Zealand context.

### Strengths

Our research has combined routine university data with equity-targeted MAPAS support programme data. As a result data misclassification is reduced (by using verified ancestry data as opposed to restricted ethnicity categories) [75,76] and both subjective and objective variables can now be explored at an individual student level [76]. In doing so, a platform for future research has been created that can link the effect of admission predictors, including equity-targeted admissions information, to academic outcomes. This research is currently underway to explore whether following MAPAS advice is associated with improved academic outcomes once students are enrolled in the University of Auckland.

Conducting a quantitative critique of the MAPAS admission process reinforces programme expectations that the advice given in December is predictive of the final recommendation given in January. This will be extremely helpful when MAPAS counsels applicants and their families as to the options likely to be available following school results. Our findings reinforce the potential for equity-targeted support programmes to conduct evidence-based decision-making when looking to enhance indigenous and ethnic minority student admission. This is important for securing ongoing funding for comprehensive equity initiatives and is of particular relevance to the MAPAS admissions process that requires significant staff contribution, faculty and government equity-funding for successful delivery [27].

This study further reinforces the notion that indigenous methodologies need not be confined to qualitative research methods [77]. Regardless, we recommend that future qualitative research should also be considered to explore applicant and whānau experiences within the MAPAS admission process in order to provide a more comprehensive understanding of the process from a participant perspective.

## Conclusion

Overall, our findings reinforce the value of using multiple selection tools, both cognitive and non-cognitive, within a comprehensive admission process for indigenous and ethnic minority applicants. The MMI, applied within an equity and indigenous cultural context, can provide important information that supports a more holistic assessment of an indigenous and ethnic minority applicant’s potential to succeed within tertiary study. Tertiary institutions committed to increasing indigenous and ethnic minority access to *and* completion of health professional studies should aim to incorporate entry processes that de-prioritise the focus on the regulation of student numbers and re-prioritise a focus on ensuring indigenous and ethnic minority student success. Differential outcomes in secondary education for Māori and Pacific students are a significant barrier of admission to health professional study at university and will continue to limit the health workforce development of indigenous and ethnic minorities in NZ unless they can be eliminated. Ethnic-specific and subject-specific solutions to improve Māori and Pacific secondary school achievement are needed. Given this context, equity-targeted admission and alternative pathways should be maintained and enhanced within tertiary institutions as a means to achieve health equity for all.

## Endnotes

^*a*^*Tangata whenua* is a Māori term that translates to ‘People of the land’. This terms refers to the indigenous status of Māori in Aotearoa NZ. ^b^The term *Pākehā* is a Māori word often used to refer to New Zealanders of European descent. This ethnic category has been used to reflect the data source i.e. the Ministry of Education, NZ. ^c^Talanoa represents the process of exchanging ideas or thinking through face-to-face conversation. ^d^The admission category of *School Leaver* refers to any MAPAS applicant applying in their final year of high school. *Alternative Admission* refers to any MAPAS applicant who is not applying as a School Leaver. ^e^*CIE (Cambridge International exams)* and *IB (International Baccalaureate)* represent alternative NZ equivalent secondary school qualification. ^f^659 out of the 918 MAPAS Interviewees had an NCEA Rank Score assessed in January (72% of the total cohort). ^g^The numbers reported are mean ± standard deviation (SD). ^h^4% (41) of the MAPAS Interview attendees had missing data for their Final MAPAS Recommendation (likely due to administrative error during the time period) resulting in 877/918 final recommendations available for analysis. ^i^OR stands for Odds Ratio. ^j^CI stands for Confidence Interval.

## Abbreviations

CertHSc: Hikitia Te Ora - Certificate in Health Sciences
CIE: Cambridge International Examination
FC: Few concerns
FMHS: Faculty of Medical and Health Sciences
GPA: Grade point average
IB: International Baccalaureate Examination
KMR: Kaupapa Māori Research
NCEA: National Certificate in Educational Attainment
NZ: New Zealand
MAPAS: Māori and Pacific Admission Scheme
MCAT: Medical College Admission Test
MMI: Multiple Mini Interview
SMC: Some or major concerns
SSO: Student Services Online
UE: University Entrance
UMAT: Undergraduate Medical and Health Sciences Admission Test
UoA: The University of Auckland
USA: United States of America
WAP: Whakapiki Ake Project
